# The family planning quotient and reproductive life index (FPQ/RepLI) tool: a solution for family planning, reproductive life planning and contraception counseling

**DOI:** 10.1186/s12978-019-0787-5

**Published:** 2019-08-19

**Authors:** Jessica M. Madrigal, Kelly Stempinski-Metoyer, Amy E. McManus, Lindsay Zimmerman, Ashlesha Patel

**Affiliations:** 1 Department of Obstetrics and Gynecology, John H. Stroger, Jr. Hospital of Cook County, 1950 W. Polk St., 7th Floor, Chicago, IL 60612 USA; 2 Division of Epidemiology and Biostatistics, School of Public Health, University of Illinois at Chicago, Chicago, IL USA; 3 Feinberg School of Medicine, Northwestern University, Chicago, IL USA; 4 Departments of Obstetrics and Gynecology, Northwestern University, Feinberg School of Medicine, Chicago, IL USA

**Keywords:** Family planning, Patient-centered care, Patient-centered counseling, Preconception care, Pregnancy planning, Reproductive goals counseling, Reproductive life plan, Reproductive life planning, Provider–patient communication

## Abstract

**Objective:**

Access to comprehensive and culturally appropriate reproductive life planning is essential to women’s health. Although many strategies and tools exist, few are designed for longitudinal use or provide visual aids. Our objective is to present the Family Planning Quotient (FPQ) and Reproductive Life Index (RepLI) (FPQ/RepLI) tool we created to facilitate the discussion of family planning and reproductive life goals between patients and providers and to provide a summary our evaluation of the tool. This tool was developed as a response to the Centers for Disease Control and Prevention’s charge of developing a tool that could help facilitate reproductive life planning by giving the patient a better understanding of their reproductive goals and trajectory.

**Study design:**

This cross-sectional evaluation of our tool took place with patients and providers at an urban, public hospital in Chicago. Patients spoke with a health educator about their sexual, gynecological, and obstetric history to complete the FPQ/RepLI tool. Our primary objective was to measure the proportion of women who indicated the tool was helpful and that they would use it to track their reproductive goals.

**Main outcome measures:**

Patients and providers completed an evaluation survey rating their satisfaction with the tool. Survey responses were summarized using frequencies and percentages.

**Results:**

During the study, 790 patients completed the evaluation.. Most patients (*n* = 725, 91.9%) agreed that the tool was helpful and that they would use it to track their reproductive goals. Fifty-five (83.5%) providers agreed that there is a need for reproductive health tools in clinical practice.

**Conclusions:**

Most agreed that the tool helped the patient communicate goals, aided in educating about contraception, and facilitated the discussion and decision-making process about available contraceptives. The tool gives patients a resource for family and reproductive goal planning. Broad dissemination amongst other medical specialties beyond obstetrics and gynecology may make reproductive life planning accessible to more women.

## Plain English summary

We created a tool for family planning called the Family Planning Quotient (FPQ) and Reproductive Life Index (RepLI) (FPQ/RepLI). We created this tool to facilitate the discussion of family planning and reproductive life goals between patients and providers. In this study we present the tool and provide a summary our evaluation of the tool. We conducted our evaluation with patients and providers at an urban, public hospital in Chicago. Patients spoke with a health educator about their sexual, gynecological, and obstetric history to complete the FPQ/RepLI tool. Following this, both patients and providers completed an evaluation survey rating their satisfaction with the tool. During the study, 790 patients completed the evaluation. Most patients (*n* = 725, 91.9%) agreed that the tool was helpful. Fifty-five (83.5%) providers agreed that there is a need for reproductive health tools in clinical practice. Most patients agreed that the tool helped the patient communicate goals, aided in educating about contraception, and facilitated the discussion and decision-making process about available contraceptives. The tool gives patients a resource for family and reproductive goal planning. Broad dissemination amongst other medical specialties beyond obstetrics and gynecology may make reproductive life planning accessible to more women.

## Introduction

Access to family planning services is fundamental to improve population health. Engaging in quality family planning gives women and their partners the ability to plan their family size and space their births, resulting in improved health outcomes for mother, child, and family [1–3]. The critical need to prioritize the health of women, their children, and their families through family planning is evidenced by the multitude of Healthy People 2020 objectives related to family planning: 1) increase the proportion of publicly funded family planning clinics that offer the full range of FDA-approved methods of contraception onsite; 2) reduce the proportion of pregnancies conceived within 18 months of a previous birth; and 3) reduce pregnancies among adolescent females [4, 5].

To meet these goals, the Centers for Disease Control and Prevention (CDC) introduced the concept of the Reproductive Life Plan (RLP) [6] to reflect a woman’s plans in terms of her desired number and timing of pregnancies in the context of her personal values and life goals. A reproductive health plan should take into account all facets of family planning, which include contraception in the preconception, interconception, and postpartum periods [7]. Initiatives such as The ONE KEY QUESTION® [8], the “Every Woman, Every Time” campaign [9], the contraceptive “vital sign” [10], Envision Sexual and Reproductive Health PATH questions [11, 12] and the Reproductive Health Self-Assessment Tool (RH-SAT) [13] incorporate questions that guide pregnancy intentions, contraceptive use, and gauge future childbearing interests. While these initiatives may facilitate conversations between patients and providers, none have been widely adopted. Existing tools may not have been adopted because of lack of evidence showing their efficacy and effectiveness in the clinical setting. There is not enough evidence that these existing tools have long term benefits to a woman’s reproductive health course. Additionally, these tools do not include visual presentations of the reproductive health plan and quantitative metric to guide patients and providers.

To address this gap, we created a visual Family Planning Quotient (FPQ) and Reproductive Life Index (RepLI) (FPQ/RepLI) tool to facilitate the discussion of family planning and reproductive life goals. The FPQ is a patient-centered family planning tool. It providers a visual representation to demonstration a women’s reproductive goals that can facilitate goal orientated management. This is a comprehensive tool incorporating biological and non-biological children, contraception, pregnancy, and infertility. Our intent was to facilitate discussion, education, and choice-making at all stages of a woman’s reproductive life and to incorporate this tool into the patient’s electronic medical record for use across our health system. The goal of our tool is to educate women about their contraceptive and reproductive options while empowering them to create reproductive life plans. The overall objective of our study was to evaluate the use of the tool with patients in the family planning and reproductive health setting. We were specifically interested in measuring the proportion of women who completed the tool and rated it as a helpful tool that they would use it to track their reproductive goals. We also describe the tool and the process of using the tool’s algorithm to effectively develop an evolving living reproductive life plan that can be incorporated into the patient’s electronic medical record.

## Methods

### Study design and evaluation

We conducted a descriptive pilot study to evaluate the FPQ/RepLI tool from 2014 to 2016 with patients and providers in the Family Planning and Reproductive Health Service clinics within the John H. Stroger, Jr. Hospital of Cook County. Patients visit these clinics for abortion services, contraception, STI screening and treatment, post-partum care, and annual gynecologic visits. During their clinical consultation, patients speak with a health educator about their sexual, gynecological, and obstetric history to capture the information to complete the FPQ/RepLI tool. Patient visits were conducted in the usual manner using the tool, and before leaving the clinic, the patient was asked to complete an evaluation of the tool and the discussion that ensued. The evaluation study was approved by the Cook County Health and Hospitals System Institutional Review Board.

To evaluate the tool, we administered an anonymous evaluation survey. Among patients, the survey was distributed during 83 clinic days spanning 26 months from July 2014 to September 2016. All patients who presented to clinic used the FPQ/RepLI tool during their encounter and were offered the evaluation survey to complete. Patients provided information on the FPQ from their visit, and rated their opinion on seven statements using a five-point Likert scale, ranging from strongly agree, to agree, neither agree nor disagree, disagree, and strongly disagree. Statements included: 1) I had discussed my reproductive life plans with my doctor prior to today’s visit; 2) Before today’s visit, my doctor knew how many children I wanted; and 3) Overall, this tool is helpful and I would use it to track my reproductive goals. Clinic providers (attending and resident physicians plus medical students) were surveyed once per month from July 2014 to July 2016. The attending physicians are permanent members of the medical staff in our health system, therefore they were surveyed one time at the start of the study after using the FPQ/RepLI tool for 1 month. The resident physicians rotate through our system on repeat because they are a set cohort that completes their four-year residency within our system. Each resident was surveyed at the end of their first monthly clinical rotation after our study began. Other residents outside of the cohort visit for a one-time rotation, in addition to medical students who rotate through our reproductive health and family planning clinics one time during their training. All of these providers were surveyed at the end of their rotation. A total of 66 providers rated their opinion on five statements, using a five-point Likert scale, ranging from strongly agree, to agree, neither agree nor disagree, disagree, and strongly disagree. Provider statements included 1) This tool improved the counseling I provided to my patient about family planning and contraception; and 2) The tool helped me to understand my patient’s reproductive plan.

Due to the anonymous nature of our survey, we used billing and administrative records to summarize the general characteristics of the patients seen in our clinic during the study period for descriptive purposes. Patients are described using frequencies and percentages for categorical, and mean and standard deviation for continuous characteristics. Survey responses were summarized using frequencies and percentages. SAS version 9.4 (Cary, NC) was used for all analyses.

### Description of the FPQ/RepLI tool

The FPQ/RepLI tool was designed for use with family planning patients to help patients and providers visualize the patient’s reproductive goals, contraceptive history, and gestational history. It follows a simple algorithm to establish future goals and facilitate plan-making. This tool was developed by looking at the FPQ, which is the current standard of care. The algorithm for this tool is based on the United States Medical Eligibility Criteria for Contraceptive Use guidelines [14], and the graphing tool is modeled off of CDC standardized pediatric growth charts [15, 16]. The tool is able to measure success based on a patient’s goals, track the implemented components required to achieve goals, and has modularity if goals change over time — essentially maintaining a living, breathing reproductive life plan for a woman. The FPQ provides a cross-sectional view while the RepLI provides a longitudinal view, both of which can be used to visually depict and quantify a woman’s reproductive life plan and assist providers in speaking to a woman’s family planning needs and interests. The FPQ/RepLI tool is a complement to other programs that outline counseling strategies such as the Balanced Counseling Strategy Plus (BCS+) [17, 18]. This tool can be used in conjunction with BCS+ to help turn the BCS+ strategy into a visual aid.

**Fig. 1:**
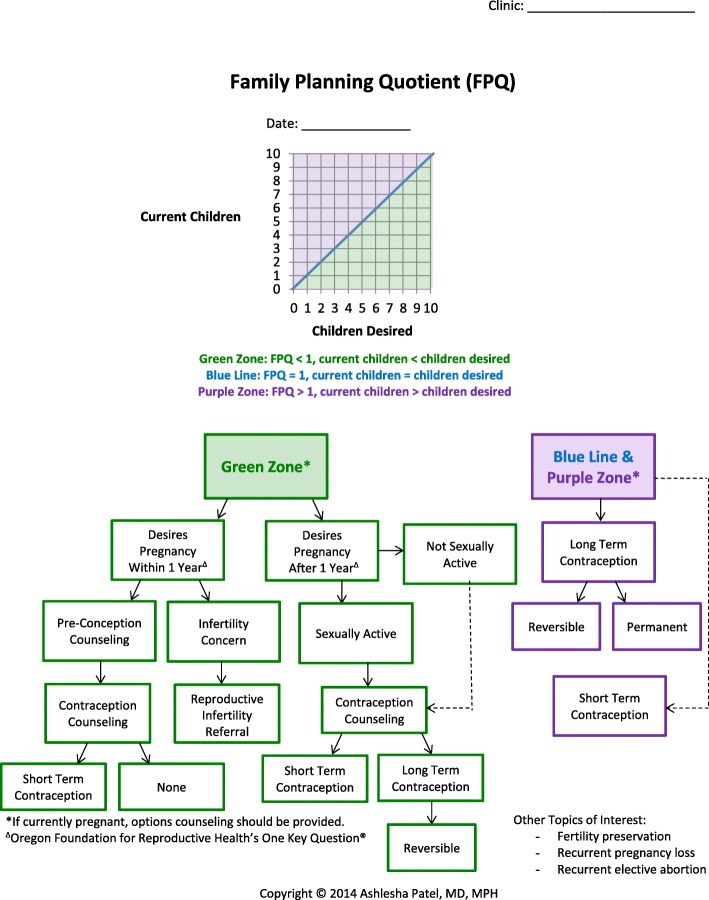
The Family Planning Quotient (FPQ) and algorithm

The FPQ/RepLI has four parts, which are a combination of visuals and text. First, the FPQ (Fig. 1 – top of page) captures the ratio of the number of living (biological and non-biological) children a woman has in the numerator, and the number of children she desires, in the denominator. A FPQ of 1, where current children *equals* desired children, indicates her goal has been achieved. This is plotted on the blue line. A FPQ of less than 1, (current children *is less than* desired children), indicates her goal has not yet been achieved. This is plotted in the “green zone”. A FPQ of greater than 1 (current children *is greater than* desired children), indicates her goal has been exceeded, which is plotted in the “purple zone”. The second part is an easy-to-follow family planning algorithm centered on the ONE KEY QUESTION® [8] (Fig. 1 – bottom of page).

**Fig. 2:**
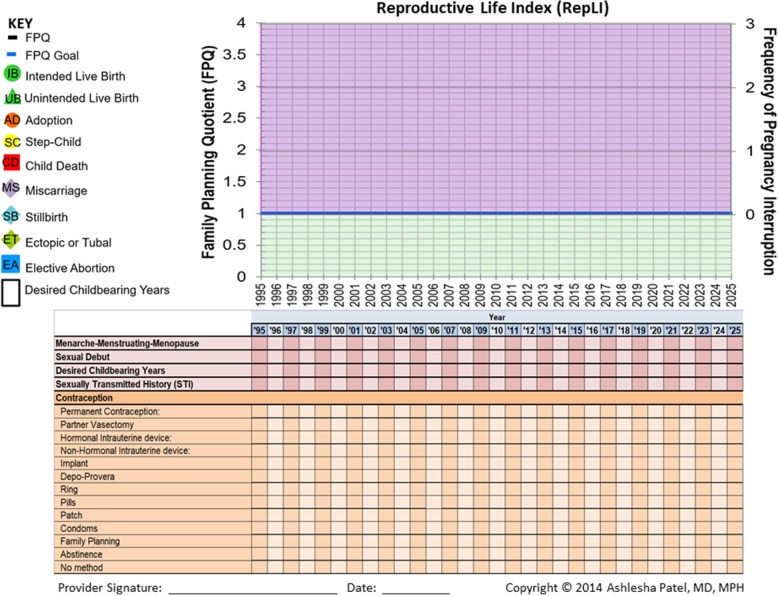
The Reproductive Life Index (RepLI)

This is followed by the RepLI (Fig. 2), which includes a longitudinal grid graphing a woman’s FPQ over time, and charting her reproductive history. RepLI then tracks other pregnancy outcomes including whether live births were intended or unintended, adopted or step-children, miscarriages, ectopic or tubal pregnancies, elective abortions, stillbirths, and child deaths. The tool also incorporates other reproductive health indicators including menarche, sexual debut, contraceptive use, and history of sexually transmitted infections (STIs).

In the setting of our publicly funded health system, most of our lay health educators are from the same racial/ethnic background as the majority of our patients. The health educators complete the FPQ/RepLI tool with the patient before the patient sees the provider. It takes approximately 5 min to administer the FPQ/RepLI tool, which is administered in English. Encounters with Spanish speaking patients are done using an interpreter, however, the FPQ/RepLI tool has not been translated into Spanish. The discussion of contraceptive needs may continue beyond the time it takes to complete all fields of the tool. In our setting the health education portion of the clinical visit where the FPQ/RepLI tool is completed takes 10 to 20 min in total, and the documented information provides an effective scaffolding upon which to build the contraceptive counseling patient encounter, as salient features of a patient’s reproductive history and goals are recorded on the sheet and provide a direction for the discussion of a contraceptive plan. The informative details include the patient’s age and history of births/miscarriages/abortions/still births; their desired childbearing years, if any; and methods of birth control used in the past and their experiences with them. This prepares the provider to have a guided goal orientated conversation with the patient about her history, pregnancy intentions, and contraception while following the algorithm in order to establish her future family planning goals. FPQs are obtained at routine patient visits and mapped over time to populate the RepLI. At the end of the visit, the completed FPQ/RepLI tool is scanned into the patient’s electronic medical record.

## Results

The FPQ/RepLI was incorporated into the clinics’ health education encounters in the beginning of 2014. During the years 2014 through 2016, the majority of our female patients were African-American (86%) or Hispanic (9%). Ages ranged from 12 to 49, with an average age of 25 years (SD 5.4 years). On average, 63% of our patients were enrolled in Medicaid, and 40% reported have attended some college or graduating college.

**Fig. 3:**
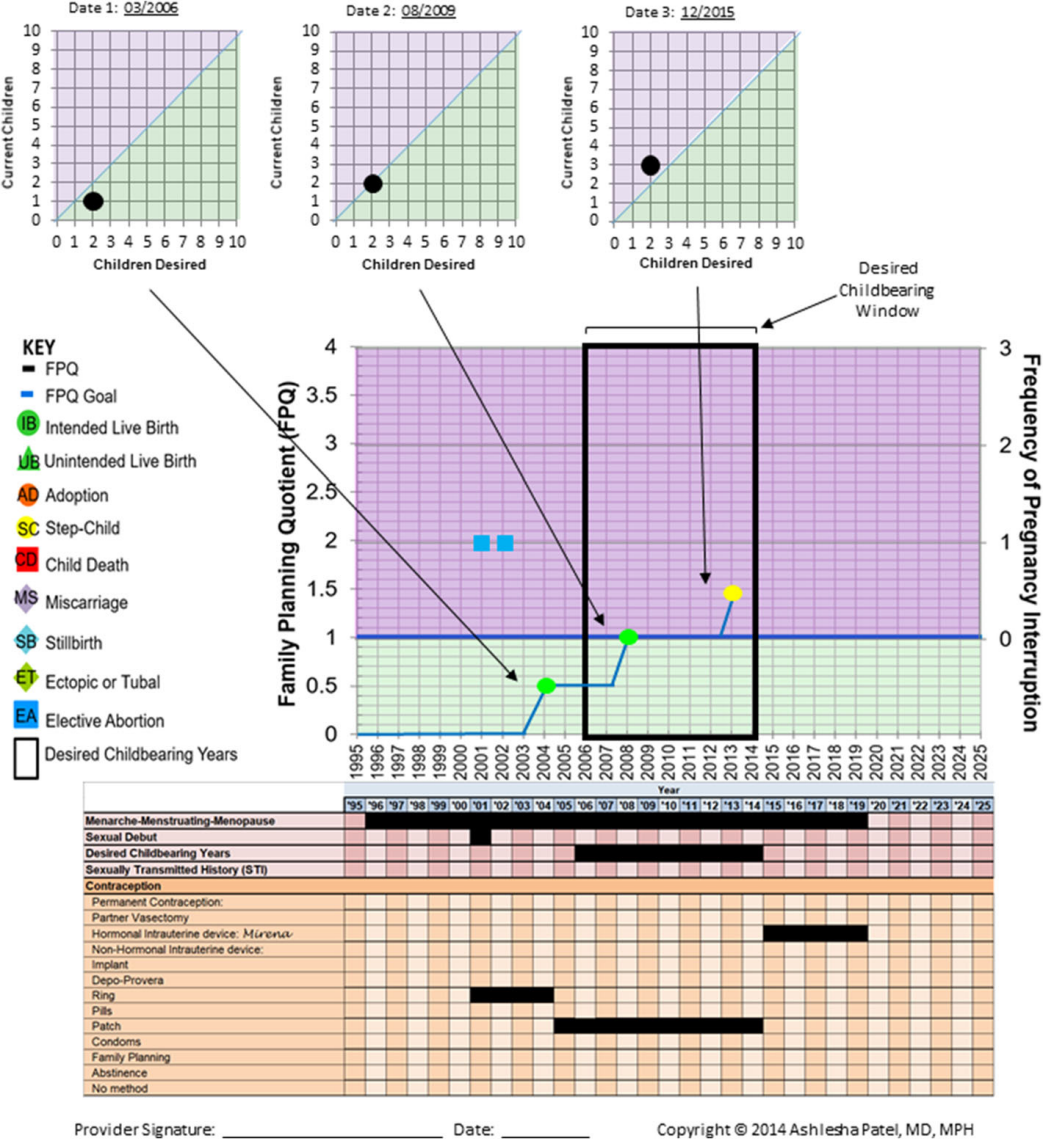
An example of a completed tool as it would be used in clinic

Figure 3 presents a patient scenario as an example of what a completed FPQ/RepLI would look like. Three different FPQs, completed at different time points, are graphed on the RepLI to show a longitudinal picture of what a woman’s reproductive life plan over time. This woman wanted two children, had two children, and then acquired a step-child through marriage, bringing her total FPQ to 3/2. The bottom half of the RepLI highlights her age at menarche, her sexual debut, her desired childbearing years, and any forms of contraception that she has/is utilizing.

As shown in Table 1, the majority of patients agreed that the tool helped communicate their personal and reproductive goals, aided in educating them about contraception, and facilitated the discussion and decision-making of available contraceptive options in clinic. Almost half of our participating patients (*n* = 362; 45.8%) indicated that their provider was unaware of their reproductive plans and goals prior to using our tool. Most patients (*n* = 725, 91.9%) agreed that the tool was helpful and that they would use it to track their reproductive goals. Though few qualitative comments were provided, one participant commented that she wanted more time with the provider to discuss contraceptive options, and another commented that she thought the RepLI portion of the tool should be larger. Of the providers surveyed, 91% (*n* = 60) agreed that the tool was useful in facilitating the conversation and understanding their patient’s reproductive plan, and 83% (*n* = 55) agreed that they saw a need for reproductive health tools like FPQ/RepLI in clinical practice and counseling.

**Table 1:** Summary of FPQ/RepLI evaluation responses among patients (*n* = 790) and providers (*n* = 66)

Patient statements	Strongly agree/agree	Neither agree nor disagree	Disagree/strongly disagree
	*n* (%)	*n* (%)	*n* (%)
Patient statements			
This tool helped me to think about my own personal goals.	742 (93.9)	42 (5.3)	6 (0.8)
This tool helped me to communicate my own personal goals to my provider.	712 (90.1)	73 (9.3)	5 (0.6)
I had thought about my reproductive life plan prior to today’s visit.	688 (87.1)	63 (8.0)	39 (4.9)
I had discussed my reproductive life plans with my doctor prior to today’s visit.	505 (64.0)	125 (15.8)	160 (20.2)
Before today’s visit, my doctor knew how many children I wanted.	276 (34.9)	152 (19.2)	362 (45.8)
Before today’s visit, my doctor knew when I wanted to have additional children (if applicable).	244 (30.9)	159 (20.1)	387 (49.0)
Overall, this tool is helpful and I would use it to track my reproductive goals.	726 (91.9)	53 (6.7)	11 (1.4)
Providers statements			
I thought this tool was useful in facilitating the conversation about reproductive health with my patient.	60 (90.9)	5 (7.6)	1 (1.5)
The tool helped me to understand my patient’s reproductive plan.	61 (92.4)	4 (6.1)	1 (1.5)
This tool helped me to focus the counseling I provided to my patient.	53 (80.3)	12 (18.2)	1 (1.5)
This tool improved the counseling I provided to my patient about family planning and contraception.	48 (72.7)	16 (24.2)	2 (3.0)
I see a need for reproductive health tools like FPQ/RepLI in clinical practice and counseling.	55 (83.3)	10 (15.2)	1 (1.5)

## Discussion

In our study, we presented a tool to facilitate reproductive life planning that works for both women who may desire pregnancy in the future and women who do not. Consensus dictates that reproductive life planning be goal-centered, personalized, collaborative, fluid, and focused on health promotion [19–21]. Our FPQ/RepLI tool reflects these attributes, and given the results of our evaluation, it has proven effective in facilitating discussions between patients and providers regarding family planning and reproductive life goals. Since reviewing the evaluation data the FPQ/RepLI has become part of our standard-of-care operating procedures in the family planning and reproductive health clinics in our public health and hospitals system. By incorporating this tool into the electronic medical record, the FPQ/RepLI becomes a permanent part of the patient’s medical history, allowing for health care providers throughout our health system and across varying specialties to access and utilize the tool in their own practices.

Numerous other strategies and tools aimed at reproductive life planning exist in the realm of family planning. My Birth Control is a contraceptive decision support tool which is used through a tablet device [22]. The initial study of My Birth Control found that compared to women who received normal contraceptive counseling, women who used My Birth Control were more likely to report complete satisfaction with their chosen method [22]. In a recent study of this tool, patients interested in beginning or changing birth control methods were randomly assigned to interact with the tool or receive usual care. Following their visits, patients were asked to fill out a survey which included questions regarding contraceptive knowledge and decision quality. Surveys were also distributed five and 7 months after receiving the new birth control method to determine continuation of the method. It was found that this intervention did not impact the likelihood of continuation, but it did positively impact contraceptive knowledge and an improved decision making experience [23]. This interactive interface was received well by patients by making them feel more involved and informed in their contraceptive decision making. In the future, the FPQ/RepLI tool could introduce this digital aspect to the study to make the experience even more interactive and to benefit the patients’ experience.

Bedsider (https://www.bedsider.org/) is another a web based contraceptive support tool aimed at women of reproductive age. After a series of focus groups were conducted, results indicated that that Bedsider was very well received by patients, but was not trusted or recommended by providers which inhibited use [24]. Similarly, an evaluation of the Smart Choices computerized tool found that patients who used the tool were pleased, but responses from providers varied in regard to how useful they found the tool with some indicating the tool had limited utility in the clinic setting [25]. This highlights the need to develop tools that are accepted as useful by both patients and their medical providers. Another study evaluated the reminder features for Bedsider to determine if these features impact contraceptive coverage and likelihood to attend scheduled medical appointments. In this study, staff were trained and encouraged to refer women to enroll in Bedsider’s special portal to receive either text message or email reminders about upcoming appointments and refill dates for their oral contraceptives. The study found that the women enrolled in reminders from Bedsider did have a high rate of return for appointments, but there was no significant change in contraceptive coverage [26].

The mobile app “miPlan” is intended to provide LARC focused contraceptive knowledge to women in the waiting room of a clinic prior to a contraceptive counseling appointment. In one study, women were randomly selected to either utilize the intervention in addition to contraceptive counseling or receive standard contraceptive counseling. Women who were assigned to miPlan had more LARC knowledge during their contraceptive counseling visit than those who did not use miPlan; however, LARC uptake was not impacted [27]. A unique feature of the app was the inclusion of short videos featuring Latina and African American women speaking about LARC. Inclusion of women from racial and ethnic minority groups into the content of the app has the potential to make women from these groups more comfortable talking about LARC, similar to when a peer health educator conducts the reproductive life planning portion of the clinical encounter. Another online questionnaire called Contraception: HeLping for WOmen’s choicE (CHLOE) was developed by gynecologists in Europe to help women better pick a contraceptive option prior to their medical appointment. It consists of 24 targeted questions to help guide a woman to contraceptive choices that might be best for their specific needs [28]. Similar to the FPQ/RepLI, CHLOE is intended to guide the discussion with a medical practitioner, not replace it. CHLOE, however, is only focused on contraception and does not cover topics across the full range of reproductive life planning.

In the setting of our clinics, the FPQ/RepLI is a convenient tool that is able to effectively cover the multidimensional nature of reproductive health discussions and decision making. The FPQ/RepLI tool contributes a more convenient, longitudinal and visual than any existing tools. Many other tools use by healthcare providers are lengthy and confusing, and require the participant to complete the tool online before interacting with anyone in the clinical setting. This tool streamlines the questions from the FPQ, and it is less laborious on the person using it. This tool also provides a longitudinal aspect that other tools do not. The longitudinal view aids in reproductive goal planning and reproductive health management by mapping benchmarks, as well as reproductive life trajectory. This tool makes contraceptive counseling simpler, modular, and more visual than before, allowing patients to better determine their needs to meet future goals. One prior study using just the FPQ portion of the tool in a group of 46 women using the Oregon Health & Science University (OHSU) Center for Women’s Health for clinical care found that 35 women agreed with the statement that the FPQ portion of the tool was helpful [29]. Differences between our evaluation and the study done in Oregon may be due to differences in the characteristics in the study samples, the intention of the clinical visit during which the tool is presented, or the fact that in our study the tool was completed and reviewed by the patient with a health educator prior to seeing the provider for discussion whereas in the OHSU study the health educator component was not implemented. Though the proportion in Oregon who agreed the FPQ tool was helpful was less than observed in our study, there is value in disseminating this tool for use in settings outside of our institution.

Our study is limited by the anonymous nature of the survey we used, in addition to the specific population of patients we serve without our health and hospital system. Our study lacks data on family planning service utilization before and after the introduction of the tool into clinical practice, limiting our ability to evaluate the full impact of the tool. There are barriers to using the tool that must also be addressed. Obtaining a patient’s obstetric and gynecological history may pose discomfort and difficulties including misunderstanding, coercion, judgement, and biases, both for the provider and the patient. To help minimize this, staff completed a tailored training on the specific questions used to populate the FPQ/RepLI tool as well as how to appropriately counsel and educate patients on family planning and reproductive goal planning, including contraception. At the time of the study, the tool was only available in English, which may have prohibited it from being useful among the small number of women served in our setting who only spoke and read Spanish. Though we utilize Spanish language interpreters in the clinical setting, in order to be useful to women who speak and read Spanish as their primary language this tool will need to be made available in Spanish.

There are also potential challenges if the encounter is not approached appropriately [30]. Pregnancy planning may not be a cultural or social norm for some women [31], and women may experience ambivalence toward an exercise to define pregnancy intentions before conception. Pregnancy ambivalence is not accounted for in the dichotomous choice of intended or unintended pregnancy in the current version of the RepLI tool. Providers should seek to deliver culturally sensitive, individually tailored counseling to encourage an effective dialogue with their patients about their reproductive life plan [5]. Lastly, the provider should emphasize to the patient that individual visits are just one cross-section of time in their reproductive life plan. The life plan thus has the ability to change from day to day.

## Conclusion

The FPQ/RepLI tool is designed to engage patients to think proactively about their reproductive goals. This tool was developed to facilitate reproductive life planning by giving the patient a better understanding of their reproductive goals and trajectory. The goal was to create a tool that utilizes the FPQ in a way that is more patient friendly and acts as a visual aid. The discussion that the RepLI facilitates covers aspects of partnership status, fertility, and sexual orientation, which are all key components of the reproductive life plan. While this tool was designed specifically for women, it has the potential to be modified for couples’ and men’s use. Furthermore, encouraging self-awareness of a woman’s reproductive health goals may facilitate further engagement in the health system and lead to participation in preventative behaviors that facilitate healthy living.

### Practice implications

Widely disseminating the tool amongst other medical specialties beyond obstetrics and gynecology, including family practice, pediatrics, and oncology/oncofertility care may make reproductive life planning accessible to more women. The tool can be modified for male and transgender patients and has the potential to give patients a resource for partner, family, and reproductive goal planning. The FPQ/RepLI is easily incorporated into electronic medical records systems, and at our own institution has evolved from a document that is scanned into the medical record to now being entered into discrete fields in the intake form used during clinical encounters. As such, the tool has the potential to become a recognizable, universal tool that can be consistently utilized across different health care systems to improve standards of reproductive healthcare.

## Abbreviations

FPQ: Family Planning Quotient
RepLI: Reproductive Life Index
